# Left Ventricular Aneurysm Repair: Off-pump Linear Plication *versus* On-pump Patch Plasty

**DOI:** 10.21470/1678-9741-2018-0366

**Published:** 2019

**Authors:** Hua Wei, Shoudong Chai, Changcheng Liu, Xinsheng Huang, Chengxiong Gu

**Affiliations:** 1 Department of Cardiac Surgery, Beijing Anzhen Hospital, Capital Medical University, Beijing, China.; 2 Beijing Institute of Heart, Lung, and Blood Vessel Diseases, Beijing, China.; 3 Department of Cardiac Surgery, Liaocheng People's Hospital, Clinical School of Taishan Medical University, Shandong, China.

**Keywords:** Left Ventricular Aneurysm, Patch Plasty, Linear Plication Repair, Outcomes

## Abstract

**Objective:**

The study aimed to compare the clinical outcomes of simplified linear plication and classic patch plasty in patients with left ventricular aneurysm (LVA).

**Methods:**

We retrospectively reviewed 282 patients undergoing LVA repair between 2006 and 2016. After propensity score matching, 45 pairs of patients receiving LVA surgery were divided into either a patch group (on-pump endoventricular patch plasty) or a plication group (off-pump linear plication). Then, their early surgical outcomes and long-term survival were compared in two matched groups.

**Results:**

The heart function improvement at discharge was similar in the two matched groups, while patients in the patch group more commonly suffered from low cardiac output syndrome (*P*=0.042) with higher proportion of intra-aortic balloon pumping assistance (*P*=0.034) than patients in the plication group. Compared with patients in the patch group, the patients in the plication group had shorter recovery times, regarding to mechanical ventilation, intensive care unit stay, and hospital stay (*P*<0.001, *P*<0.001, and *P*=0.001, respectively). No significant difference was found in the long-term survival (*P*=0.62).

**Conclusions:**

Off-pump linear plication presented acceptable results in terms of early outcomes and long-term survival. For high-risk patients, the simplified LVA repair technique may be an option.

**Table:** 

Abbreviations, acronyms & symbols				
AMI	= Acute myocardial infarction		LVA	= Left ventricular aneurysm
CABG	= Coronary artery bypass grafting		LVAD	= Left ventricular aneurysm dimensions
COPD	= Chronic obstructive pulmonary disease		LVEDVI	= Left ventricular end-diastolic volume index
CPB	= Cardiopulmonary bypass		LVEF	= Left ventricular ejection fraction
IABP	= Intra-aortic balloon pumping		LVESVI	= Left ventricular end-systolic volume index
ICU	= Intensive care unit		NYHA	= New York Heart Association
LAD	= Left anterior descending branch		RV	= Right ventricle
LIMA	= Left internal mammary artery		SD	= Standard deviation
LV	= Left ventricle		SV	= Saphenous vein

## INTRODUCTION

Left ventricular aneurysm (LVA) secondary to acute myocardial infarction (AMI) can lead to a poor prognosis. Heart failure^[1]^ is the most common cause of death in patients with LVA duo to severe left ventricle (LV) remodeling. Surgical repair is still the optimal therapy for LVA. The Dor procedure (endoventricular circular patch plasty)^[2]^ has been advocated as a classic surgical technique with a more physiologic geometric reconstruction. But this procedure must be performed with ventriculotomy and cardiopulmonary bypass (CPB) assist. And extensive myocardial incision could cause additional myocardial damage, possibly impairing the cardiac function and increasing the operative risk.

Recently, the linear plication of LVA without ventriculotomy and CPB assist has been accepted as a simplified surgical technique^[3,4]^. However, the most appropriate LVA repair technique remains a matter of controversy. In this retrospective study, we compared off-pump linear plication with on-pump patch plasty regarding to early surgical outcomes and long-term survival.

## METHODS

### Patient population

From 2006 to 2016, 316 patients suffering from severe coronary disease and LVA underwent myocardial revascularization and LVA repair. Thirty-four patients who also presented mural thrombosis of LV, ventricular arrhythmias, or valve disease were excluded, remaining the 282 patients who were recruited in the study. According to the strategy of the LVA reconstruction, 97 patients underwent the Dor procedure (patch group), and the others underwent off-pump linear plication repair (plication group). Baseline characteristics, surgical outcomes, and survival information were collected from medical records and patients' follow-up visits. The study was carried out in accordance with the Ethical Guideline of the Committee on Human Experimentation of our institution, and the informed consent was obtained from the patients about the experimentation.

To reduce the selection bias, the differences in the distribution of baseline characteristics between the patch group and the plication group were eliminated by matching on the propensity score. And the propensity score was calculated using the logistic regression model based on the following variables: age, gender, hypertension, diabetes mellitus, dyslipidemia, chronic obstructive pulmonary disease (COPD), prior cerebrovascular disease, renal dysfunction, New York Heart Association (NYHA) class, left ventricular ejection fraction (LVEF), number of diseased arteries, LVA location, and left ventricular aneurysm dimensions (LVAD) (mmxmm) measured by the apical two-chamber view of LV in transthoracic echocardiogram. Based on the calculated propensity score, matching between the two groups was achieved through a propensity score matching algorithm. Finally, 45 pairs of patients were successfully matched in a 1:1 manner (area under the receiver operating characteristic curve = 0.764; Hosmer-Lemeshow statistic = 0.2).

### Surgical Techniques

All patients underwent standard procedural protocol involving median sternotomy under general anesthesia and harvesting of the left internal mammary artery (LIMA) and saphenous vein (SV). The decision on the LVA repair technique was based on the size of the aneurysmal sac during surgery. Dor procedure was performed in case of a large scar with marked aneurysmal sac, whereas off-pump linear plication was preferred in case of a small scar.

In the patch group, CPB was established following the routine moderate systemic hypothermia (esophageal 26°C-32°C). After cross-clamping the aorta, myocardial protection was achieved by antegrade perfusion and graft perfusion with cold, blood-infused cardioplegia. After the distal anastomosis of the traditional coronary artery bypass grafting (CABG), the LVA was repaired using the Dor procedure (endoventricular scar resection and circular patch plasty). Dor procedure details refer to a previous study^[5]^. Once the ventricular repair was completed, the proximal anastomosis was created on the site of antegrade perfusion.

**Fig. 1:**
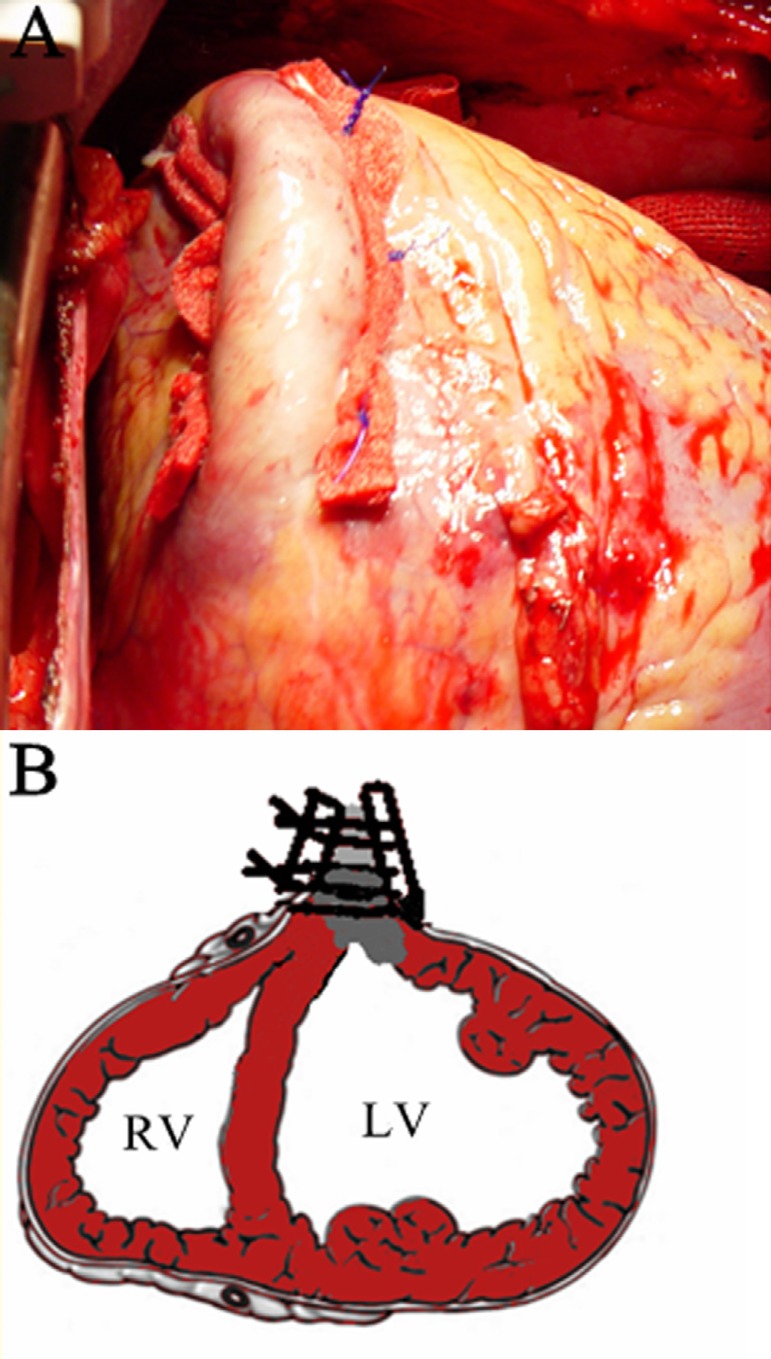
Off-pump plication repair technique of left ventricular aneurysm. (A) Linear plication was performed with Teflon felt successively in a horizontal mattress fashion. (B) Schematic diagram showed the linear plication. LV=left ventricle; RV=right ventricle

In the plication group, off-pump CABG was performed firstly. Following, the LVA and the transitional zone of viable myocardium and thinned scar tissue were confirmed by visual inspection and palpation. Then, LVA was isolated in the transitional zone and 2-0 Surgipro843 sutures were passed through one strip of Teflon felt, the rim of the aneurysm, and the opposite strip of the Teflon felt, successively, in a horizontal mattress fashion. Plication was designed to remove as much nonfunctioning thin wall as possible while restoring the ventricular size and shape using the linear closure (Figure 1).

### Follow-Up

The follow-up was conducted via telephone contact with patients or revisits to our outpatient department. Primary endpoint was cardiac death. All patients were followed up until mid-September 2017.

### Statistical Analysis

Statistical analysis was performed using an extensively admissive software program, the SAS software (version 9.4; SAS Institute Inc., Cary, NC, USA). Data were presented as means ± standard deviation (SD) for continuous variables and as frequencies and percentages for categorical variables. The continuous variables were analyzed using Wilcoxon rank-sum analysis. Chi-squared test and Fisher's exact test were applied for the comparison of categorical variables. Log-rank (Mantel-Cox) survival curves were made to compare the long-term survivals of the two surgical interventions. All the *P* values < 0.05 were considered statistically significant.

## RESULTS

### Baseline Characteristics

Baseline data were showed in Table 1. The two groups had different demographics and concomitant diseases. Patients in the patch group suffered from more severe left ventricular remodeling than those in the plication group (LVEF: 33.50±7.35% *versus* 38.32±7.86%; LVAD: 51mmx43mm *versus* 38mmx32mm, respectively; *P*<0.01). However, patients in the plication group were older than those in the patch group (62.32±9.23 *versus* 59.58±8.26, respectively; *P*=0.015). Compared with the patch group, the diabetes mellitus was more common in the plication group, 40.00% *versus* 27.83%, respectively (*P*=0.043). Furthermore, the plication group had a higher proportion of high-risk patients with severe concomitant diseases than the patch group, with respect to peripheral vascular diseases, COPD, and prior cerebrovascular diseases (24.86% versus 14.43%, 12.97% *versus* 4.12%, and 16.22% *versus* 7.22%, respectively; *P*<0.05). After the propensity score matching, the characteristic distribution of any demographics, hemodynamic data, and severe concomitant diseases in the two groups had a non-significant difference. The details were showed in Table 2.

**Table 1:** Baseline characteristics of the two groups.

Variables	Patch group (n=97)	Plication group (n=185)	*P*
Age (years)	59.58±8.26	62.32±9.23	0.015
Males (%)	77 (79.38)	152 (82.16)	0.57
Hypertension (%)	42 (43.29)	79 (42.70)	0.923
Diabetes mellitus (%)	27 (27.83)	74 (40.00)	0.043
Dyslipidemia (%)	48 (49.48)	88 (47.56)	0.76
LVEF (%)	33.50±7.35	38.32±7.86	<0.01
NYHA class			0.269
I or II	18 (18.55)	45 (24.32)	
III or IV	79 (81.45)	140 (75.68)	
Number of diseased vessels			0.42
1 vessel	4 (4.12)	6 (3.24)	
2 vessels	14 (14.43)	35 (18.92)	
3 vessels	79 (81.45)	144 (77.84)	
Aneurysm location (%)			0.516
Anterior	76 (78.35)	141 (76.22)	
Apical	18 (18.56)	37 (20.00)	
Posterior	3 (3.09)	7 (3.78)	
LVAD (mmxmm)	51x43	38x32	<0.01
Peripheral vascular disease	14 (14.43)	46 (24.86)	0.042
COPD	4 (4.12)	24 (12.97)	0.034
Prior cerebrovascular disease	7 (7.22)	30 (16.22)	0.033
Renal dysfunction	5 (5.26)	12 (6.49)	0.655

COPD=chronic obstructive pulmonary disease; LVAD=left ventricular aneurysm dimensions; LVEF=left ventricular ejection fraction; NYHA=New York Heart Association

**Table 2:** Baseline characteristics of the two matched groups.

Variables	Patch group (n=45)	Plication group (n=45)	*P*
Age (years)	60.33±8.52	61.13±8.17	0.65
Males (%)	37 (82.22)	36 (80.00)	0.788
Hypertension (%)	18 (40.00)	19 (42.22)	0.83
Diabetes mellitus (%)	13 (28.89)	11 (24.44)	0.634
Dyslipidemia (%)	22 (48.89)	24 (53.33)	0.673
LVEF (%)	35.53±6.94	37.19±7.42	0.276
NYHA class			0.598
I or II	8 (17.76)	10 (22.22)	
III or IV	37 (82.24)	35 (77.78)	
Number of diseased vessels			0.739
2 vessels	4 (9.89)	6 (13.33)	
3 vessels	41 (91.11)	39 (86.67)	
Aneurysm location (%)			
Anterior	40 (88.89)	42 (93.33)	0.714
Apical	5 (11.11)	3 (6)	
LVAD (mmxmm)	42x36	40x35	0.393
Peripheral vascular disease	7 (15.56)	8 (17.78)	0.777
COPD	1 (2.22)	1 (2.22)	1.0
Prior cerebrovascular disease	3 (6.67)	3 (6.67)	1.0
Renal dysfunction	2 (4.44)	1 (2.22)	1.0

COPD=chronic obstructive pulmonary disease; LVAD=left ventricular aneurysm dimensions; LVEF=left ventricular ejection fraction; NYHA=New York Heart Association

### Operative Characteristics and Early Prognosis

No significant statistical difference was found between the two matched groups in terms of number of grafts and LIMA-left anterior descending branch (LAD) anastomosis. The early mortality at discharge was 4.44% in the patch group, but there was no early death in the plication group. In addition, patients in patch group more commonly suffered from low cardiac output syndrome (42.22% *versus* 22.22%, *P*=0.042) with higher proportion of intra-aortic balloon pumping assistance (37.78% *versus* 17.78%, *P*=0.034) than those in the plication group. Major postoperative complications including stroke and acute renal failure were similar between the two matched groups, but patients in the plication group had shorter recovery time, regarding to mechanical ventilation, intensive care unit (ICU) stay, and hospital stay (*P*<0.001, *P*<0.001, and *P*=0.001, respectively) than those in the patch group. The details were showed in Table 3. Patch plasty and linear plication repairs both improved cardiac geometry and function remarkably. The echocardiography indicated that no significant differences between the two surgical techniques were founded with respect to LVEF, left ventricular end-diastolic volume index (LVEDVI), and left ventricular end-systolic volume index (LVESVI) (Table 4).

**Table 3:** Intra- and post-operative outcomes in the two matched groups.

Variables	Patch group (n=45)	Plication group (n=45)	*P*
Number of grafts	3.73±1.01	3.67±0.88	0.765
LIMA to LAD (%)	36 (80.0)	35 (77.78)	0.796
IABP support	17 (37.78)	8 (17.78)	0.034
Surgical death	2 (4.44)	0	0.153
Low cardiac output syndrome	19 (42.22)	10 (22.22)	0.042
Stroke	3 (6.67)	1 (2.22)	0.306
Acute renal failure	3 (6.67)	0	0.078
Mechanical ventilation time (hour)	27.83±10.31	19±8.25	<0.001
ICU stay (day)	3.98±2.16	2.47±1.35	<0.001
Hospital stay (day)	14.83±5.02	11.14±3.78	0.001

IABP=intra-aortic balloon pumping; ICU=intensive care unit; LAD=left anterior descending branch; LIMA=left internal mammary artery

**Table 4:** Pre- and post-operative 3 months echocardiography parameters in the two matched groups.

Variables	Patch group			Plication group		
	Preoperative	Postoperative	*P*	Preoperative	Postoperative	*P*
LVEDVI (ml/m^2^)	137.12±40.92	101.87±21.36	<0.001	132.25±42.36	98.93±23.15	<0.001
LVESVI (ml/m^2^)	104.35±50.67	64.39±31.24	<0.001	102.17±51.82	62.57±32.31	<0.001
LVEF (%)	35.53±6.94	49.46±9.24	<0.001	37.19±7.42	48.15±10.13	<0.001

LVEF=left ventricular ejection fraction; LVEDVI=left ventricular end-diastolic volume index; LVESVI=left ventricular end-systolic volume index

### The Long-Term Survival

**Fig. 2:**
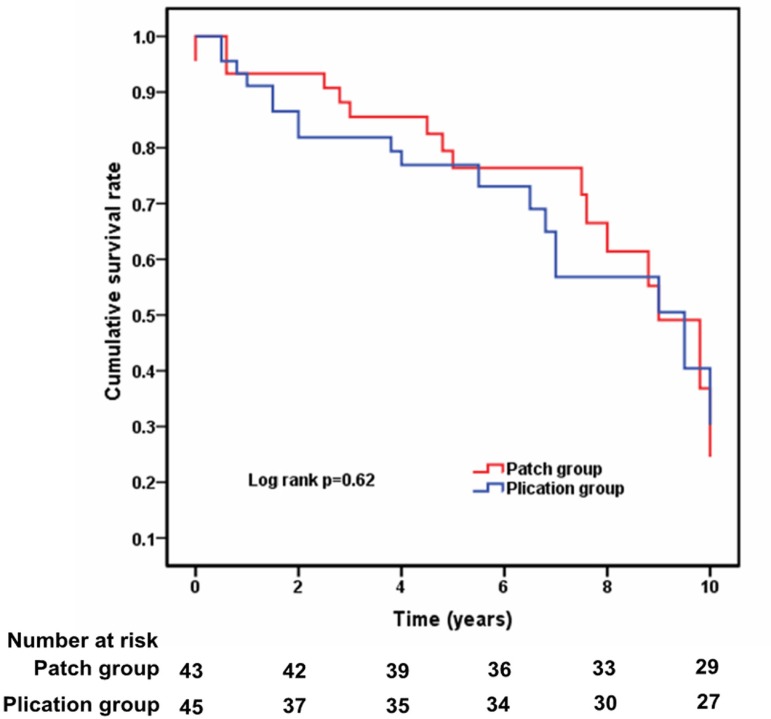
Estimated Mantel-Cox survival curves for two matched patients.

Follow-up was complete for the two matched groups. The mean duration of follow-up was 5.5 years (1 year-10.3 years). Patients' overall survival at 1 year, 5 years, and 10 years were 93.33%, 80%, and 64.44%, respectively, in the patch group *versus* 91.11%, 75.56%, and 60%, respectively, in the plication group. The survival curve indicated that cumulative survival rate in the two matched groups had no significant difference (Figure 2).

## DISCUSSION

The normal shape of LV is a prolate ellipsoid^[6]^. In post-infraction LVA, akinetic or dyskinetic aneurysmal segments distort the LV ellipsoid geometry, which leads to LV dilatation and spherical remodeling. The main purpose of LVA surgery is to exclude the infracted region and to revert the ventricular dilatation, spherically remodeling it to a more ellipsoid geometry. Dor procedure^[7]^ and off-pump linear plication^[8]^ have been proven to ensure clinical benefits by improving LV geometry and reducing ventricular volume. Our results with good surgical outcomes were similar to those from previous studies. And the law of Laplace^[9]^ also explains the clinical benefits from LVA repair. As noted before, the two surgical techniques reduced the LV wall tension remarkably by reducing the radius LV cavity, which benefits the patients with LVA repair in the following aspects: reducing the consumption of oxygen at the cardiac level; improving ventricular fibers orientation, and thus the contractile efficiency; and enabling the heart to do the same amount of work, but with a lower expenditure of energy^[10]^.

Although the Dor procedure has been accepted as a classic and effective technique for LVA repair, the limitations of the technique should be considered. Firstly, the endocardial patch plasty excluded an akinetic or dyskinetic region and replaced it with an akinetic patch, which may be counterproductive to reconstruct the LV. Secondly, the circular patch maybe affects the realignment of non-diseased myocardial fibers^[11]^. Thirdly, on-pump extensive resection of endocardial scar maybe affects postoperative recovery duo to extra myocardial damage and inherently negative effects of CPB. Our results showed that the mean time of mechanical ventilation, ICU stay, and hospital stay were longer in the patch group than in the plication group.

Comparing with on-pump patch plasty, off-pump linear plication may be a less invasive repair strategy. Off-pump linear plication maintains the integrity of the ventricular wall and avoids ventriculotomy, and thus possible myocardial damage. Also, it is performed on beating hearts in order to facilitate visual identification of noncontractile zones of LVA, which ensures to absolutely isolate the akinetic or dyskinetic region from the viable myocardium. But the more challenging aspects of off-pump linear plication are intraventricular thrombi, the non-removal of scarred septum, and concomitant ischemic mitral regurgitation. Fortunately, large-sized LVA combining with mural thrombosis or valve diseases are much less prevalent now than in the past. The probable reason is the use of timely and aggressive postischemic treatments, including thrombolysis, acute percutaneous coronary intervention, as well as other medical anti-thrombosis and anti-heart failure therapies. So, off-pump linear plication, as a relatively simple and less invasive technique, may be promising for LVA with small dimensions, not combining mural thrombi and valve diseases.

Furthermore, CABG is generally considered as an important part of LVA surgical treatments, irrespective of repair techniques. CABG not only improves ischemia, probably enhancing global contraction of LV, but also prevents angina pectoris and ventricular arrhythmias. And the clinical outcomes might be more likely to be achieved with total revascularization and LIMA to the LAD^[12]^. In recent years, off-pump CABG, an effective surgical technique, has been demonstrated to benefit particularly the high-risk population, because this technique avoids the manipulation of the CPB and eliminates complications secondary to CPB^[13,14]^. Off-pump linear plication combined with off-pump CABG may be an acceptable strategy for patients with postinfraction LVA. And Huang et al^[3]^ demonstrated that patients with LVA could benefit from the combined intervention (off-pump CABG and linear plication) with acceptable symptomatic relief and long-term survival.

### Limitations

This study has some limitations. The study was susceptible to inherent bias from retrospective nature. But propensity score matching on baseline characteristics resulted in equivalent risk profiles between the two groups. The cohort was small because the subjects belonged to a highly selected subgroup. And patients with LVA concomitant with valve diseases and mural thrombosis did not meet the study criteria.

## CONCLUSION

LVA repair concomitant with surgical revascularization can be performed with relatively low operative mortality while markedly improving the clinical status of the patients. Off-pump linear plication obtained the acceptable results in terms of early outcomes and long-term survival. For high-risk patients, the simple LVA repair technique may be an option.

**Table:** 

Authors' roles & responsibilities	
HW	Carried out the study including collecting data and follow-up; wrote the article; final approval of the version to be published
SC	Carried out the study including collecting data and follow-up; final approval of the version to be published
CL	Analyzed the data; final approval of the version to be published
XH	Carried out the study including collecting data and follow-up; final approval of the version to be published
CG	Designed the study and revised the article; final approval of the version to be published
